# Supplementary figures and images for: Psychosocial interventions and their effectiveness on quality of life among elderly persons living with HIV in Africa South of the Sahara: Systematic review and meta -analysis protocol
Source: PLoS One. 2023 Sep 20;18(9):e0291781. doi: 10.1371/journal.pone.0291781 (PMC10511069; doi:10.1371/journal.pone.0291781)

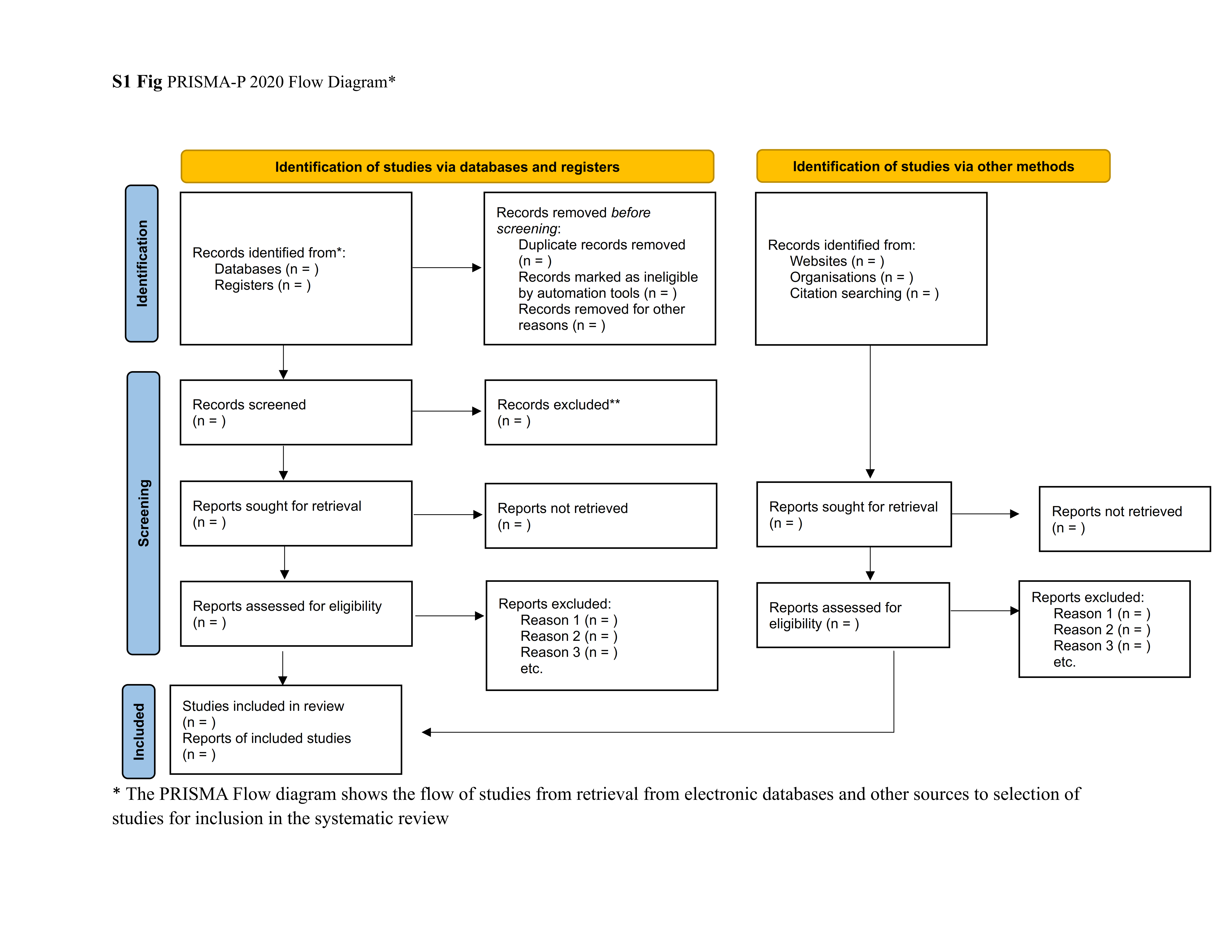

Supplement: S1 Fig — The PRISMA Flow diagram shows the flow of studies from retrieval from electronic databases and other sources to selection of studies for inclusion in the systematic review. (TIF) [file pone.0291781.s001.tif]
