# Supplementary material for: Psychosocial interventions and their effectiveness on quality of life among elderly persons living with HIV in Africa South of the Sahara: Systematic review and meta -analysis protocol
Source: PLoS One. 2023 Sep 20;18(9):e0291781. doi: 10.1371/journal.pone.0291781 (PMC10511069; doi:10.1371/journal.pone.0291781)
Supplement: S2 Table — (PDF) [file pone.0291781.s003.pdf]

S2 Table: Hoy Risk of Bias Tool

Risk of bias item	Criteria for answers
**External validity**	
1. Was the study's target population **<u>a close representation</u>** of the population of interest in relation to relevant variables, e.g. age, sex, occupation, health status or other?	• **Yes (LOW RISK):** The study's target population was a close representation of the national population.<br>• **No (HIGH RISK):** The study's target population was clearly NOT representative of the national population.
2. Was the sampling frame a **<u>true or close representation</u>** of the target population?	• **Yes (LOW RISK):** The sampling frame was a true or close representation of the target population.<br>• **No (HIGH RISK):** The sampling frame was NOT a true or close representation of the target population.
3. Was some form of **random selection** used to select the sample, OR, was a census undertaken?	• **Yes (LOW RISK):** A census was undertaken, OR, some form of random selection was used to select the sample (e.g. simple random sampling, stratified random sampling, cluster sampling, systematic sampling).<br>• **No (HIGH RISK):** A census was NOT undertaken, AND some form of random selection was NOT used to select the sample.
4. Did the study avoid inappropriate exclusions?	• **No (LOW RISK)**<br>• **Yes (HIGH RISK)**
**Internal validity**	
5. Was an acceptable case definition used in the study?	• **Yes (LOW RISK):** An acceptable case definition was used.<br>• **No (HIGH RISK):** An acceptable case definition was NOT used.
6. Is the study method for measuring drug prescription shown to have **<u>reliability and validity (if necessary)</u>**? i.e. is there an opportunity for misclassification	• **Yes (LOW RISK):** The method is shown to have minimal misclassification potential<br>• **No (HIGH RISK):** The method is NOT shown to have minimal misclassification potential
7. Was the **<u>same mode of data collection</u>** used for all subjects?	• **Yes (LOW RISK):** The same mode of data collection was used for all subjects.<br>• **No (HIGH RISK):** The same mode of data collection was NOT used for all subjects.
8. Were the **<u>numerator(s) and denominator(s)</u>** for the parameter of interest appropriate?	• **Yes (LOW RISK):** The paper presented appropriate numerator(s) AND denominator(s) for the parameter of interest.<br>• **No (HIGH RISK):** The paper did present numerator(s) AND denominator(s) for the parameter of interest but one or more of these were inappropriate.
**Summary item on the overall risk of study bias**	
• **LOW RISK OF BIAS:** Further research is very unlikely to change our confidence in the estimate.<br>• **MODERATE RISK OF BIAS:** Further research is likely to have an important impact on our confidence in the estimate and may change the estimate.<br>• **HIGH RISK OF BIAS:** Further research is very likely to have an important impact on our confidence in the estimate and is likely to change the estimate.	
