# Supplementary material for: Psychosocial interventions and their effectiveness on quality of life among elderly persons living with HIV in Africa South of the Sahara: Systematic review and meta -analysis protocol
Source: PLoS One. 2023 Sep 20;18(9):e0291781. doi: 10.1371/journal.pone.0291781 (PMC10511069; doi:10.1371/journal.pone.0291781)
Supplement: Attachment — Submitted filename: Psychosocial Interventions QoL HIV SR-final.docx [file pone.0291781.s004.docx]

**Psychosocial Interventions and their Effectiveness on Quality of Life Among Elderly Persons Living with HIV/AIDS in Sub-Saharan Africa: Systematic Review Protocol**

Marijanatu Abdulai^1,2^, David Owiredu^1,3^, Isaac Boadu^4^, Philip Teg-Nefaah Tabong^5^, Bismark Sarfo^1^, Harriet Affran Bonful^1^, Adolphina Addo- Lartey^1^, Kwadwo Owusu Akuffo^6^, Anthony Danso-Appiah^1,3*^

**Author affiliation**

^1^ Department of Epidemiology and Disease Control, School of Public Health, University of Ghana, Legon, Accra

^2^ National AIDS/STI Control Programme, Public Health Division, Ghana Health Service, Accra

^3^ Centre for Evidence Synthesis and Policy, School of Public Health, University of Ghana, Legon, Accra

^4^ Department of Population, Family and Reproductive Health, School of Public Health, University of Ghana, Legon, Accra

^5^ Department of Social and Behavioural Science, School of Public Health, University of Ghana, Legon, Accra

^6^ Department of Vision Sciences, Kwame Nkrumah University of Science and Technology, Kumasi, Ghana

**Author email addresses**

Marijanatu Abdulai: [mabdulai@nacp.org.gh](mailto:mabdulai@nacp.org.gh)

David Owiredu; [evasupreme@gmail.com](mailto:evasupreme@gmail.com)

Isaac Boadu: [isaacboadu62@gmail.com](mailto:isaacboadu62@gmail.com)

Philip Teg-Nefaah Tabong: ptabong@ug.edu.gh

Bismark Sarfo: [sbismark@yahoo.com](mailto:sbismark@yahoo.com)

Kwadwo Owusu Akuffo: [koakuffo@gmail.com](mailto:koakuffo@gmail.com)

Harriet Affran Bonful: [habonful@ug.edu.gh](mailto:habonful@ug.edu.gh)

Adolphina Addo Lartey: aaddo.lartey@gmail.com

Anthony Danso-Appiah: [adanso-appiah@ug.edu.gh](mailto:adanso-appiah@ug.edu.gh)

***Corresponding author**

Dr. Anthony Danso-Appiah, DSc, PhD; Centre for Evidence Synthesis and Policy, School of Public Health, University of Ghana, P. Box LG13, Legon, Accra, Ghana.

Email:	[adanso-appiah@ug.edu.gh](mailto:adanso-appiah@ug.edu.gh); ;[tdappiah@yahoo.co.uk](mailto:tdappiah@yahoo.co.uk)

Tel:	+233 269083789; +44 7958721789

**ORCID IDs**

Marijanatu Abdulai: 0000-0002-1189-5976

David Owiredu: 0000-0002-4503-1850

Isaac Boadu: 0000-0003-2703-7391

Philip Teg-Nefaah Tabong: 0000-0001-9445-1643

Bismark Sarfo: 0000-0001-9445-1643

Kwadwo Owusu Akuffo: 0000-0001-6683-249X

Harriet Affran Bonful: 0000-0003-0069-3241

Adolphina Addo Lartey: 0000-0001-8852-7189

Anthony Danso-Appiah: 0000-0003-1747-0060

**Word count**

Main Text Word Count:

Tables: 1

Figures: 1

Additional files: 3

1. Additional File 1: PRISMA-P Checklist
2. Additional File 2: PRISMA Flow Diagram
3. Additional file 4: Hoy Risk of Bias Tool

**Abstract**

**Background:** The number of elderly people living with HIV (EPLHIV) has increased significantly as a result of antiretroviral treatment (ART) and this has brought about a variety of psychosocial challenges that have an impact on their quality of life (QoL). Various psychosocial interventions have been tried or implemented in Sub-Saharan Africa (SSA) to improve QoL of EPLHIV. However, there is paucity of data on the types and effectiveness of these interventions. This systematic review, therefore, aims to explore available psychosocial interventions in SSA and their effectiveness in improving the QoL of EPLHIV.

**Methods:** We will search PubMed, PsycINFO, LILACS, Cochrane Library, Google Scholar, HINARI, Africa Journals Online, Scopus and Web of Science to retrieve publications on psychosocial interventions implemented to improve QoL of EPLHIV from 1^st^ January 2010 to 30^th^ September 2023 without language restrictions. Also, supplementary sources such as conference proceedings, preprint repositories, databases of dissertations, as well as WHO and governmental databases can be explored for additional studies. For unpublished studies, trial registries and experts would be contacted, and reference lists of retrieved papers will be manually searched. Retrieved studies will be deduplicated using Mendeley and exported to Rayyan. At least two reviewers will independently select studies, extract data and assess the quality of the included studies using validated tools. Dichotomous outcomes data will be assessed and reported as odds ratio (OR) or risk ratio (RR) and for continuous outcomes, mean difference (MD) will be used; all reported with their 95% confidence interval (CI). Heterogeneity will be explored graphically by inspecting the overlapping of CIs and assessed quantitatively using the I^2^ statistic.

**Expected outcomes:** This systematic review will be the first to rigorously identify psychosocial intervention on QoL of EPLHIV in SSA and assess their effectiveness with the aim to provide regional and country- specific data that will inform the selection and implementation of appropriate and socially acceptable policies across countries in SSA. Key findings of the review are expected to contribute critical evidence on availability, types and effectiveness of psychosocial interventions for improving quality of life of vulnerable elderly persons in SSA living with HIV. Furthermore, the review will explore any variation and possible correlates of psychosocial interventions by age, sex, CD4 count (if available), setting and geographic location within SSA that will provide healthcare professionals with reliable evidence, with the ultimate goal of inspiring countries in SSA to adopt innovative interventions to improve HIV/AIDS care.

**Systematic Review Registration:** The systematic review protocol has been registered in the International Prospective Register for Systematic Reviews (PROSPERO), with registration ID **CRD42021278218.**

**Keywords:** Psychosocial Interventions, Quality of Life, HIV/AIDS, Systematic Review, Elderly people living with HIV, sub-Saharan Africa.

**Background**

Human Immune deficiency virus (HIV) and Acquired Immune Deficiency Syndrome (AIDS) remain global public health challenge claiming many lives despite significant effort to reduce the spread of the infection [1,2]. Over 38.4 million people were living with HIV/AIDS in 2021, with 1.5 million new infections and 650 000 HIV- related deaths globally, of which 36.7 million were adults (15 years and above), with Sub-Saharan Africa (SSA) accounting for 25.4 million deaths (66.1%) [3,4].

The introduction of antiretroviral therapy (ART) has resulted in a significant reduction in HIV-related morbidities and mortalities whilst increasing life expectancy among people living with HIV (PLHIV)[5]. Antiretrovirals are highly effective in suppressing the replication of HIV, reducing the viral load in the body. This helps slow the progression of HIV disease, preserving immune function and preventing further damage to the immune system [6]. Empirical evidence suggests that the number of PLHIV on lifelong ART has tripled from 7.5 million in 2010 to 27.5 million in 2020, of which a higher proportion (61%) live in East and Southern Africa [7,8]. Despite the benefits of ART, HIV infection and related psychosocial effects still impact the quality of life (QOL) of PLHIV significantly, especially the elderly who are already overwhelmed with the challenges of ageing and associated comorbidities [9,10].

Psychosocial well-being, a multidimensional concept comprising psychological, social, and other subjective mental states, remains a big challenge for PLHIV generally, but critically among EPLHIV [11]. Globally, the prevalence of depression, anxiety and mood disorders accounts for about 33% among PLHIV [12,13]. A systematic review of PLHIV in sub-Saharan Africa found pooled estimates of anxiety and depression to be 32% and 9%, respectively [14,15]. Psychological symptoms usually lead to nonadherence to ART, poor rating of QoL and increased HIV-related deaths [16]. In North America and Europe, evidence showed that a positive HIV test result alone and the perception of life changes associated with a life-threatening, chronic illness led to episodes of depression and anxiety [13,17,18].

It was not surprising that the 2021 Technical report of the WHO emphasized the increased risk of mental health problems among PLHIV, particularly EPLHIV, which usually leads to less motivation to adhere to treatment, increased risk behaviours, and decreased engagement with HIV prevention measures [19,20]. Therefore, a top priority of the 2021-2026 Global AIDS Strategy has been the integration of mental health and psychosocial support with HIV services, especially those led by communities to help improve the QoL of PLHIV [20,21].

**The rationale for this systematic review**

People living with HIV are more susceptible to HIV-related neurocognitive dysfunction due to mitochondrial damage, similar to a common occurrence in elderly persons [22]. They may be accompanied by undesirable physical and psychological changes affecting QoL [23,24]. Elderly people living with HIV are particularly vulnerable because their age is a vital precursor of adverse mitochondria outcomes. Psychosocial interventions such as cognitive behavioural therapy (CBT), supportive interventions, meditation and stress management to improve QoL have been developed for EPLHIV in developed countries with some positive outcomes [25–28]. Despite the evidence suggesting the need to focus on improving the QoL of EPLHIV because of their increased vulnerability and needs, there is paucity of data in SSA about the availability and effectiveness of psychosocial interventions for improving the QoL of this unique group [29–32]. Undoubtedly, psychosocial interventions targeted to EPLHIV are critical to achieving SDG 3 [33], however, this is simply a neglected issue and not on the policy agenda of most countries across SSA [33,34].

Although several systematic reviews on the impact of psychosocial intervention on QoL among the elderly exist but these have focused on chronic diseases such as cancers [32,35], chronic heart failure and type II diabetes mellitus [22,36,37] but there is little or no evidence on psychosocial interventions targeting the QoL of EPLHIV in SSA. Given the high number of EPLHIV in SSA and the trend pointing to a further increase, it is of utmost importance to identify and assess, through systematically synthesized data, available psychosocial interventions and their effectiveness in Sub-Saharan Africa to inform the sound and context-specific policies for improving QoL of EPLHIV [13,20,36].

This systematic review aims to document available psychosocial interventions to improve the QoL of EPLHIV in SSA and assess their effectiveness. In particular, the review will answer the following specific questions; what psychosocial interventions on QoL are available in SSA? 2) which of them are effective in improving QoL of EPLHIV? and 3) what are the correlates of QoL EPLHIV in the Sub-Saharan African context?

**Methods**

This protocol has been prepared in line with standard systematic review manuals and reported per the reporting guidance provided in the Preferred Reporting Items for Systematic Reviews and Meta-Analyses Protocol (PRISMA-P) statement [38] (Additional file 1). The full review will be reported per the PRISMA guidelines [39] and the PRISMA flow (Additional file 2).

**Patient and public involvement**

The review questions and outcome measures have been developed collaboratively with the relevant patients and public involvement. They are informed by their priorities, experience, and preferences per the Guidance for Reporting Involvement of Patients and the Public (GRIPP2) checklist. The review findings will be shared with relevant wider patient communities, who will also be involved in disseminating the results.

**Criteria for including studies for inclusion in this systematic review**

**Type of studies**

Randomized Controlled Trials (RCTs), quasi-experimental studies, and observational studies, including cohort, case-control, and cross-sectional studies that reported on psychosocial interventions and quality of life of elderly persons living with HIV within sub-Saharan Africa, will be eligible for inclusion. If sufficient RCTs are obtained, the review will be restricted to only RCTs. Reviews, opinions, commentaries, case reports and case series will not be eligible for inclusion in this review. Whilst reviews are not eligible for inclusion, we will review them to identify potentially relevant primary studies missed by our searches. For a multi-country study involving SSA countries, we will extract the studies conducted in SSA for inclusion in this review. If the study reported a country or regional-level estimate, it would not be eligible for inclusion as such studies do not have a well-defined source population. In cases where the results of a multi-country study have been lumped together and there is no way of disaggregating the data, such studies will not be included.

**Participants**

The population for this systematic review is elderly persons aged 50 years and older receiving ART for HIV and living in SSA. The Centre for Disease Control CDC has defined elderly PLHIV as those aged 50+ years and not 65+ as classified for the general population by the WHO. This CDC classification for older HIV patients is that PLHIV age faster than the non-HIV population [40,41]. The HIV status of participants to be included should have been determined by prequalified testing approach, an algorithm set out by the WHO and adopted by member countries [42]. Participants should have been registered at a recognized health facility providing antiretroviral treatment services and receiving their routine ARVs. HIV-positive clients who are not on ARVs, are pregnant or breastfeeding, will be excluded. No restrictions will be placed on participants' gender, ethnicity, or other demographic characteristics.

**Intervention**

All psychosocial interventions to improve QoL among EPLHIV will be eligible for inclusion. Interventions will include, but are not limited to, the following: 1) social support, 2) psychotherapy, 3) psychoeducation and counselling, and 4) cognitive behavioural therapy [43,44].

Psychosocial interventions for QoL improvement administered individually, in groups, or a combination of the two through mobile technology, online platforms, healthcare professionals, and social workers [45] will be eligible for inclusion in the review. Psychosocial interventions not specifically targeting participants' QoL will be excluded.

**Comparator**

The comparator will be elderly people aged 50 years and above living with HIV and AIDS in SSA receiving ARVs (or standard treatment for HIV with no additional psychosocial interventions targeted to improving their QoL.

**Outcomes**

***Primary outcomes***

The primary outcome of this review is "improvement in quality of life". There are about 150 instruments available for measuring QoL [46]. However, this review will assess the five main QoL measurement scales, namely, the Quality of Life Scale (QoLS) by Flanagan 1978 [47], the McGill Quality of Life Questionnaire was developed by Cohen et al in 1996 and expanded in 2019 [48], the Health-Related Quality of life questionnaire developed by the CDC in 2000, the World Health Organization Quality of Life Instrument [49] and the Global Quality of Life Scale developed in 1996 [50]. These measurements have been selected because of their psychometric properties and wide application across geographic locations and contexts [51].

***Secondary outcomes***

- Availability of tools in SSA
- Correlates of psychosocial interventions on QoL

**Adverse events**

All adverse events reported by the included studies, especially from the RCTs, will be summarized in this systematic review.

**Searches for the identification of studies**

**Electronic searches**

We will search all relevant electronic databases for relevant primary studies on psychosocial interventions to improve the quality of life of elderly persons living with HIV. The primary databases to be searched are PubMed, LILACS, CINAHL and Cochrane Library. The searches will be done from 1^st^ January 2010 to 30^th^ September 2023, without language restrictions. Other sources such as Google Scholar, African Journals Online, JSTOR, SCOPUS, HINARI, and dissertation and preprint databases will also be searched. The key search terms and concepts to be used are psychosocial intervention, quality of life, HIV, AIDS, elderly people living with HIV, EPLHIV, antiretroviral therapy and sub-Saharan Africa used with all applicable synonyms and alternative terms. All 48 sub-Saharan African countries based on the World Bank classification will be included individually as search terms in the search (see Table 1 for the search strategy developed for PubMed, which will be adapted for the other databases).

**Other sources to be searched**

Reference lists of relevant studies and published systematic reviews on the subject will be manually searched for potentially eligible studies. Additionally, experts in the field will be contacted for completed but unpublished studies.

**Table 1** Search Strategy developed for PubMed (to be adapted for the other databases)

| Search | Query | Results |
| --- | --- | --- |
|  | Search: (#7) AND (#8) |  |
|  | Search: (((((((((((((((((((((((((((((((((((((((((((((((("sub-Saharan Africa") OR (SSA)) OR (Angola)) OR (Benin)) OR (Botswana)) OR ("Burkina Faso")) OR (Burundi)) OR (Cameroon)) OR ("Cape Verde")) OR ("Central African Republic")) OR (Chad)) OR (Comoros)) OR (Congo)) OR ("Cote d'Ivoire")) OR (Djibouti)) OR ("Equatorial Guinea")) OR (Ethiopia)) OR (Gabon)) OR ("The Gambia")) OR (Ghana)) OR (Guinea)) OR ("Guinea-Bissau")) OR (Kenya)) OR (Lesotho)) OR (Liberia)) OR (Madagascar)) OR (Malawi)) OR (Mali)) OR (Mauritania)) OR (Mauritius)) OR (Mozambique)) OR (Namibia)) OR (Niger)) OR (Nigeria)) OR (Rwanda)) OR ("Sao Tome and Principe")) OR (Senegal)) OR (Seychelles)) OR ("Sierra Leone")) OR (Somalia)) OR ("South Africa")) OR (Sudan)) OR (Swaziland)) OR (Tanzania)) OR (Togo)) OR (Uganda)) OR (Zaire)) OR (Zambia)) OR (Zimbabwe) |  |
|  | Search: (#5) AND (#6) |  |
|  | Search: (((((((adults[Title/Abstract]) OR (elderly[Title/Abstract])) OR (aged[Title/Abstract])) OR ("older people"[Title/Abstract])) OR ("matured people"[Title/Abstract])) OR ("matured persons"[Title/Abstract])) OR ("grown-ups"[Title/Abstract])) OR ("older persons"[Title/Abstract]) |  |
|  | Search: (#3) AND (#4) |  |
|  | Search: (((("HIV/AIDS"[Title/Abstract]) OR ("Human immunodeficiency virus"[Title/Abstract])) OR ("Acquired immune deficiency syndrome"[Title/Abstract])) OR (HIV[Title/Abstract])) OR (AIDS[Title/Abstract]) |  |
|  | Search: (#1) AND (#2) |  |
|  | Search: (((((((("quality of life"[Title/Abstract]) OR (QOL[Title/Abstract])) OR (wellbeing[Title/Abstract])) OR (well-being[Title/Abstract])) OR (welfare[Title/Abstract])) OR (happiness[Title/Abstract])) OR ("standard of living"[Title/Abstract])) OR ("living standard"[Title/Abstract])) OR (satisfaction[Title/Abstract]) |  |
|  | Search: (((((Psychosocial Interventions[Title/Abstract]) OR (programs[Title/Abstract])) OR (programmes[Title/Abstract])) OR (strategy[Title/Abstract])) OR (strategies[Title/Abstract])) OR (innovations[Title/Abstract]) |  |

**Managing the search output and selecting studies**

All results from the various searches will be collated and deduplicated using the Mendeley reference manager (<https://www.mendeley.com/reference-manager/library/all-references>). The results will then be exported to Rayyan QCRI, a systematic review web app: <https://rayyan.ai/terms> for screening and selection of studies. The titles and abstracts of articles will be screened independently by at least two review team members using the study selection flow chart developed from the inclusion/exclusion criteria (Figure). Where a full-text article cannot be retrieved or is not accessible from the online databases, the team will contact the primary authors. If they fail to help, we will seek assistance from the University of Ghana Library through their inter-library policy. A PRISMA flow chart [52] will be provided to show details of the study flow through the study selection process. Any disagreements will be resolved through discussion between the reviewers.

**Data extraction and management**

Data will be extracted using a pretested form developed in Microsoft Excel. The following study characteristics will be extracted: title, author, year of publication, study area/setting, the country where the study was conducted, study design, sample size, age of participants, and ART status. Then, pertinent data on the interventions and outcomes, such as the type of psychosocial intervention and scale used in measuring QoL, will be collected. Data extracted during the piloting stage will be checked and compared between the data extractors for accuracy and consistency before finally applying the form to the rest of the studies. In the case of more than two reviewers doing the data extraction, 10% of the included studies will be sampled and extracted independently by an experienced reviewer in the team as a quality check. Where necessary, the data extraction form will be updated based on experience learned and all changes made will be documented transparently. Any disagreements between reviewers will be resolved through discussion.

**Assessment of quality of the included studies**

The Cochrane risk of bias tool available in Cochrane Handbook and Review Manager V5.4 will be used to assess the risk of bias in each of the included RCTs in six domains: sequence generation, allocation concealment, blinding (investigators, outcome assessors, and participants), incomplete outcome data, selective outcome reporting and other sources of bias. For each domain, we will make a judgment of 'low risk of bias, 'high risk of bias or 'unclear risk of bias. All the risk of bias domains will be considered to assign each study a low, high, or unclear risk of bias. The Cochrane tool will be used in addition to the quality assessment tool for prevalence studies developed by Hoy et al. (2012) [53] that assesses the risk of bias in observational studies on four domains: selection bias, non-response bias, measurement bias, and bias related to data analysis. This will be done independently by at least two review team members. For each domain, a judgment of 'low risk of bias,' high risk of bias or unclear risk of bias will be made. The overall quality of evidence contributed by each study will be classified as 'low risk of bias',' high risk of bias or unclear risk of bias. Any discrepancy will be resolved through discussion between the reviewers.

**Plan for data synthesis**

The findings from the included studies will be narratively summarized by the PICOS elements, including the type and characteristics of the participants/population, type of studies, type of psychosocial interventions, and outcomes of interest in the characteristics of the included studies. The quantitative data will be analyzed using Stata version 15.0 (Stata Corp LLC, Texas, USA) and Review Manager v5.4. Dichotomous data will be presented as odds ratio (OR) or risk ratio (RR) and for continuous outcome data, mean difference (MD) will be used with all expressed with their respective confidence intervals (CIs). The following process will be used: firstly, standard deviations for the study-specific estimates, where applicable, will be determined from point estimates and the appropriate denominators assuming a binomial distribution. Then the magnitude of heterogeneity between included studies will be assessed quantitatively using the index of heterogeneity (I² statistics). The I^2^ values of 25%, 50%, and 75% are considered to represent low, moderate and significant heterogeneity, respectively [54]. The significance of heterogeneity will be determined by the p-value of the I² statistics, and a p-value of <0.05 will be considered evidence of heterogeneity. For studies with moderate to significant heterogeneity, the random effects model will be used to obtain a pooled estimate of the outcome, and if heterogeneity is low, a fixed-effect model will be used.

**Heterogeneity and subgroup analysis**

Where the heterogeneity detected is significantly high, a subgroup analysis will be performed to detect the possible sources mainly around the characteristics of the PICOS elements (P-participants/population, I-intervention, C-control/comparator, O-outcomes and S-study type). Specially, we will assess heterogeneity around potential confounding variables such as age, study setting (hospital or community-based), sex, and geographical location (Central, East, Southern and West Africa). Sub-group analysis for RCTs will be done carefully in order not to break the randomization code of the trials.

**Handling missing and incomplete data**

We will attempt to extract sufficient data from all included studies. Where data on some pertinent variables are missing, the original authors of the primary studies will be contacted to see if they can provide the missing data. Where original authors are unable to provide the requested information or cannot be reached, we will not compute. Instead, the potential impact of missing data on the findings of the review will be addressed in the discussion section.

**Sensitivity analysis**

Sensitivity analysis, the process of testing the robustness of the results of a systematic review [55],

will be used to re-analyze the data to determine if the results are sensitive to specific review elements [56,57]. The domains to be considered in the sensitive analysis include the quality of the included studies, sample size and the meta-analysis technique applied.

**Grading level of evidence**

The Grading of Recommendations, Assessment, Development, and Evaluations (GRADE) approach will be used to assess the overall quality of evidence generated from the review for each outcome. The pooled data of each study will be evaluated against the five GRADE considerations; risk of bias, imprecision, inconsistency, indirectness, and publication bias and will be graded as high, high, moderate, low and very low levels of evidence accordingly [58,59]. High-quality evidence is interpreted as further research is very unlikely to change the confidence in the estimate of effect, while a grading of very low quality implies an estimate of effect is very doubtful.

**Discussion**

The introduction of ARTs means that people living with HIV will increasingly live longer and face age-related challenges and HIV-related psychosocial problems, including quality of life. This review is the first to attempt to rigorously summarize evidence on psychosocial interventions for QoL of EPLHIV in SSA to provide country and regional- specific estimates of the effect of psychosocial interventions for improving QoL of EPLHIV that will inform the implementation of context and culturally sensitive policies. The review uses rigorous approaches to address issues relating to external validity (generalizability) using the PICOS to succinctly define the characteristics of the participants, interventions, outcomes and types of studies. It uses transparent and robust methods (as specified in the methods) to generate reliable evidence from a very comprehensive search strategy that will attempt to retrieve all potentially relevant studies from a wide range of study designs such as randomized controlled trials (RCTs), cohort, case-control and cross-sectional studies to gather evidence. Subgroup analysis will be performed to explore whether psychosocial interventions vary according to age, sex, setting and geographic location across SSA. It will also explore socio-demographic characteristics, ART histories and interventions sought among EPLHIV. It is anticipated that these review findings will provide healthcare professionals with evidence-based information regarding the effectiveness of psychosocial interventions in enhancing the QoL of EPLHIV. Moreover, the review has the potential to inspire countries in SSA to adopt innovative interventions to improve HIV and AIDS care based on reliable evidence.

SUGGEST POSSIBLE LIMITATIOSN AND HOW YOU WILL OVERCOME THEM

SUGGEST IMPLICATIONS OF THE ANTICIPATED STUDY FINDINGS

**Acknowledgements**

This systematic review was prepared as part of the capacity-building initiative of the Centre for Evidence Synthesis and Policy, University of Ghana and Africa Communities of Evidence Synthesis and Translation (ACEST) that jointly train in evidence synthesis and translation across countries in Africa and Low and Middle Income Countries (LMICs). MA was mentored by ADA.

**List of Abbreviations**

| AIDS | Acquired Immune Deficiency Syndrome |
| --- | --- |
| ART | Antiretroviral treatment |
| ARVs | Antiretrovirals |
| CBT | Cognitive Behavioural Therapy |
| CDC | Centers for Disease Control and Prevention |
| CI | Confidence Interval |
| EPLHIV | Elderly persons living with HIV |
| HIV | Human Immune deficiency virus |
| HRQOL | Health-Related Quality of Life |
| LMICs | Low- and Middle-Income Countries |
| MD | Mean Difference |
| OoL | Quality of Life |
| OR | Odds Ratio |
| PICOS | Patient/Population. Intervention. Comparison, Outcomes |
| PLHIV | Persons living with HIV |
| PRISMA | Preferred Reporting Items for Systematic Review and Meta-Analysis |
| PROSPERO | Prospective Register for Systematic Reviews |
| QoLS | Quality of Life Scale |
| RCT | Randomized Controlled Trials |
| RR | Risk Ratio |
| SDGs | Sustainable Development Goals |
| SSA | Sub-Saharan Africa |
| UGCESP | University of Ghana Centre for Evidence Synthesis and Policy |
| WHO | World Health Organisation |

**Ethics and dissemination:** Ethics approval is not required for this review as the work will be carried out on published and unpublished completed primary studies. The findings will be disseminated in a related peer-review journal and presented at conferences. Findings from the systematic review will also serve as a guide for policymakers in decision-making across countries in SSA.

**DECLARATIONS**

**Ethical Approval and consent to Participate**

Not applicable

**Consent for publication**

Not applicable

**Availability of Data and Materials**

Not applicable

**Competing Interests**

The review team members declare no competing interests.

**Funding**

The review of this protocol development has not received any funding.

**Contribution of Authors**

This protocol has been designed and conceptualized by MA, DO, IB and ADA

The search strategy was developed by MA, DO, BS, AAL, and ADA

The manuscript was drafted by MA, KOA, DO, PTN, H AB, and ADA with inputs from all authors

The manuscript was critically reviewed by MA, KOA, PTN, H AB, DO, IB, BS, AAL and ADA

Agreement to be accountable for the accuracy of all aspects of the work; MA, DO, IB, PTN, BS, KOA, HAB, AAL and ADA

Supervision by ADA

**References**

1.	Hu H, GuyTaylor, Chen Q. HIV Education: Raising Awareness and Increasing Knowledge. In: n: Wu, Z, Wang, Y, Detels, R, Bulterys, M, McGoogan, J (eds) HIV/AIDS in China. 2020. p. 171–82.

2.	Dzah SM, Tarkang EE, Lutala PM. Knowledge, attitudes and practices regarding HIV/AIDS among senior high school students in Sekondi-Takoradi metropolis, Ghana. African J Prim Heal Care Fam Med. 2019;11(1):1–11.

3.	UNAIDS. Global HIV Statistics. Fact Sheet 2021. 2022;(June):1–3.

4.	Oladimeji KE, Dzomba A, Adetokunboh O, Zungu L, Yaya S, Ter Goon D. Epidemiology of multimorbidity among people living with HIV in sub-Saharan Africa: a systematic review protocol. BMJ Open. 2020;10(12):e036988.

5.	Tsuda H, Koga M, Nojima M, Senkoji T, Kubota M, Kikuchi T, et al. Changes in survival and causes of death among people living with HIV: Three decades of surveys from Tokyo, one of the Asian metropolitan cities. J Infect Chemother. 2021;27(7):949–56.

6.	Carrington M, Alter G. Innate immune control of HIV. Cold Spring Harb Perspect Med. 2012;2(7):a007070.

7.	Autenrieth CS, Id EJB, Stelzle D, Mallouris C, Mahy M, Ghys P. Global and regional trends of people living with HIV aged 50 and over : Estimates and projections for 2000 – 2020. NIH Public Access Author Manuscr. 2020;1–11.

8.	Gakhar H, Kamali A, Holodniy M. Health-related quality of life assessment after antiretroviral therapy: A review of the literature. Drugs. 2013;73(7):651–72.

9.	Sok P, Gardner S, Bekele T, Globerman J, Seeman M V, Greene S, et al. Unmet basic needs negatively affect health-related quality of life in people aging with HIV: results from the Positive Spaces, Healthy Places study. BMC Public Health. 2018;18(1):1–10.

10.	Tavares MC de A, Leal MCC, Marques AP de O, Zimmermann RD. Social support for the elderly with HIV/Aids: an integrative review. Rev Bras Geriatr e Gerontol. 2019;22.

11.	Kumar C. Psychosocial well-being of individuals. Qual Educ. 2020;676–86.

12.	Nyongesa MK, Nasambu C, Mapenzi R, Koot HM, Cuijpers P, Newton CRJC, et al. Psychosocial and mental health challenges faced by emerging adults living with HIV and support systems aiding their positive coping: a qualitative study from the Kenyan coast. BMC Public Health. 2022;22(1):1–20.

13.	Yasamy MT, Dua T, Harper M, Saxena S. a Growing Concern. Ment Heal older people. 2013;4.

14.	van Luenen S, Garnefski N, Spinhoven P, Spaan P, Dusseldorp E, Kraaij V. The Benefits of Psychosocial Interventions for Mental Health in People Living with HIV: A Systematic Review and Meta-analysis. AIDS Behav. 2018;22(1):9–42.

15.	Bernard C, Dabis F, de Rekeneire N. Prevalence and factors associated with depression in people living with HIV in sub-Saharan Africa: a systematic review and meta-analysis. PLoS One. 2017;12(8):e0181960.

16.	Busi AN, Nsoh M, Otieno MO, Ndeso SA, Halle-ekane GE, Busi-bageorgetownedu AN, et al. Evaluation of quality of life and associated factors among HIV patients on antiretroviral therapy in North West region of Cameroon. 2021;21:8–17.

17.	Gonzalez JS, Batchelder AW, Psaros C, Safren SA. Depression and HIV/AIDS treatment nonadherence: a review and meta-analysis. J Acquir Immune Defic Syndr. 2011;58(2).

18.	Adams C, Zacharia S, Masters L, Coffey C, Catalan P. Mental health problems in people living with HIV: changes in the last two decades: the London experience 1990–2014. AIDS Care. 2016;28(sup1):56–9.

19.	Bantjes J, Kagee A. Common mental disorders and psychological adjustment among individuals seeking HIV testing: a study protocol to explore implications for mental health care systems. Int J Ment Health Syst. 2018;12:1–10.

20.	World Health Organization. Integration of mental health and HIV interventions. 2021;1–72.

21.	Remien RH, Patel V, Chibanda D, Abas MA. Integrating mental health into HIV prevention and care: a call to action. J Int AIDS Soc. 2021;24(Suppl 2).

22.	Spies G, Asmal L, Seedat S. Cognitive-behavioural interventions for mood and anxiety disorders in HIV: a systematic review. J Affect Disord. 2013;150(2):171–80.

23.	Schank M, Zhao J, Moorman JP, Yao ZQ. The impact of HIV-and ART-induced mitochondrial dysfunction in cellular senescence and aging. Cells. 2021;10(1):174.

24.	Nasi M, De Biasi S, Gibellini L, Bianchini E, Pecorini S, Bacca V, et al. Ageing and inflammation in patients with HIV infection. Clin Exp Immunol. 2017;187(1):44–52.

25.	Gerans R, Waluyo A. A Psychosocial Intervention for Mental Health of People with HIV (PLWH): A Literature Review. Int J Nurs Heal Serv. 2019;2(3):45–57.

26.	Scofield D, Moseholm E. HIV-related stigma and health-related quality of life in women living with HIV in developed countries: a systematic review. AIDS Care. 2022;34(1):7–15.

27.	Fong BYF, Law VTS, Leung TCH, Lo MF, Ng TKC, Yee HHL. Ageing and Sustainable Development Goal 3 in Hong Kong. Sustainable Development Goal 3. 2021. 22–43 p.

28.	Negin J, Cumming RG. HIV infection in older adults in sub-Saharan Africa: extrapolating prevalence from existing data. Bull World Health Organ. 2010;88:847–53.

29.	Catalan J, Tuffrey V, Ridge D, Rosenfeld D. What influences quality of life in older people living with HIV? AIDS Res Ther. 2017;14(1):1–10.

30.	Siedner MJ. Aging, health, and quality of life for older people living with HIV in Sub-Saharan Africa: a review and proposed conceptual framework. J Aging Health. 2019;31(1):109–38.

31.	Mwangala PN, Mabrouk A, Wagner R, Newton CRJC, Abubakar AA. Mental health and well-being of older adults living with HIV in sub-Saharan Africa: a systematic review. BMJ Open. 2021;11(9):e052810.

32.	Gabriel I, Creedy D, Coyne E. A systematic review of psychosocial interventions to improve quality of life of people with cancer and their family caregivers. Nurs open. 2020;7(5):1299–312.

33.	Bekker L-G, Alleyne G, Baral S, Cepeda J, Daskalakis D, Dowdy D, et al. Advancing global health and strengthening the HIV response in the era of the Sustainable Development Goals: the International AIDS Society—Lancet Commission. Lancet. 2018;392(10144):312–58.

34.	Woltz PC, Chapa DW, Friedmann E, Son H, Akintade B, Thomas SA. Effects of interventions on depression in heart failure: a systematic review. Hear Lung. 2012;41(5):469–83.

35.	Graham CD, Gouick J, Krahé C, Gillanders D. A systematic review of the use of Acceptance and Commitment Therapy (ACT) in chronic disease and long-term conditions. Clin Psychol Rev. 2016;46:46–58.

36.	Hanlon P, Daines L, Campbell C, McKinstry B, Weller D, Pinnock H. Telehealth interventions to support self-management of long-term conditions: a systematic metareview of diabetes, heart failure, asthma, chronic obstructive pulmonary disease, and cancer. J Med Internet Res. 2017;19(5):e172.

37.	Wing EJ. HIV and aging. Int J Infect Dis. 2016;53:61–8.

38.	Moher D, Shamseer L, Clarke M, Ghersi D, Liberati A, Petticrew M, et al. Preferred reporting items for systematic review and meta-analysis protocols (PRISMA-P) 2015 statement. Syst Rev. 2015;4(1):1–9.

39.	Page MJ, McKenzie JE, Bossuyt PM, Boutron I, Hoffmann TC, Mulrow CD, et al. The PRISMA 2020 statement: an updated guideline for reporting systematic reviews. Int J Surg. 2021;88:105906.

40.	McMillan JM, Krentz H, Gill MJ, Hogan DB. Managing HIV infection in patients older than 50 years. CMAJ. 2018;190(42):E1253–8.

41.	Althoff KN, Smit M, Reiss P, Justice AC. HIV and ageing: improving quantity and quality of life. Curr Opin HIV AIDS. 2016;11(5):527.

42.	WHO. Consolidated guidelines on HIV prevention, testing, treatment, service delivery and monitoring: recommendations for a public health approach. Geneva: World Health Organization. 2021. 594 p.

43.	Okonji EF, Mukumbang FC, Orth Z, Vickerman-Delport SA, Wyk B V. Psychosocial Support Interventions for Improved Adherence and Retention in ART care for Young People Living with HIV: A Scoping Review. 2020;

44.	van der Kop ML, Muhula S, Patel A, Thabane L, Awiti P, Kyomuhangi L, et al. Gender differences in health-related quality of life at the time of a positive HIV test–a cross-sectional study in a resource-poor, high prevalence setting in Nairobi, Kenya. AIDS Care. 2018;30(4):493–9.

45.	Leslie M, Beatty L, Hulbert-Williams L, Pendrous R, Cartwright T, Jackson R, et al. Web-Based Psychological Interventions for People Living With and Beyond Cancer: Meta-Review of What Works and What Does Not for Maximizing Recruitment, Engagement, and Efficacy. JMIR cancer. 2022;8(3):e36255.

46.	Celestine N. 5 Quality of Life Questionnaires and Assessments T. Body Brain. 2022;1–36.

47.	Burckhardt CS, Anderson KL, Archenholtz B, Hägg O. The Flanagan quality of life scale: Evidence of construct validity. Health Qual Life Outcomes. 2003;1(1):1–7.

48.	Cohen SR, Mount BM, Tomas JJN, Mount LF. Existential well‐being is an important determinant of quality of life: Evidence from the McGill quality of life questionnaire. Cancer Interdiscip Int J Am Cancer Soc. 1996;77(3):576–86.

49.	De Vries J, Van Heck GL. The World Health Organization Quality of Life assessment instrument (WHOQOL-100): validation study with the Dutch version. Eur J Psychol Assess. 1997;13(3):164–78.

50.	Hyland ME, Sodergren SC. Development of a new type of global quality of life scale, and comparison of performance and preference for 12 global scales. Qual Life Res. 1996;5:469–80.

51.	Tesemma AL, Abate MG, Abebo ZH, Madebo WE. Determinants of poor quality of life among adults living with hiv and enrolled in highly active antiretroviral therapy at public health facilities of arba minch town administration in southern ethiopia. HIV/AIDS - Res Palliat Care. 2019;11:387–94.

52.	Moher D Tetzlaff J, Altman Dg LA. PRISMA 2009 Flow Diagram. The PRISMA statement. 2009.

53.	Hoy D, Brooks P, Woolf A, Blyth F, March L, Bain C, et al. Assessing risk of bias in prevalence studies: modification of an existing tool and evidence of interrater agreement. J Clin Epidemiol. 2012;65(9):934–9.

54.	Thorlund K, Imberger G, Johnston BC, Walsh M, Awad T, Thabane L, et al. Evolution of heterogeneity (I2) estimates and their 95% confidence intervals in large meta-analyses. PLoS One. 2012;7(7):e39471.

55.	Mowbray FI, Manlongat D, Shukla M. Sensitivity Analysis: A Method to Promote Certainty and Transparency in Nursing and Health Research. Can J Nurs Res. 2022;54(4):371–6.

56.	Marušić MF, Fidahić M, Cepeha CM, Farcaș LG, Tseke A, Puljak L. Methodological tools and sensitivity analysis for assessing quality or risk of bias used in systematic reviews published in the high-impact anesthesiology journals. BMC Med Res Methodol. 2020;20(1):1–10.

57.	Mathur MB, VanderWeele TJ. Sensitivity analysis for publication bias in meta‐analyses. J R Stat Soc Ser C Appl Stat. 2020;69(5):1091.

58.	Granholm A, Alhazzani W, Møller MH. Use of the GRADE approach in systematic reviews and guidelines. Br J Anaesth. 2019;123(5):554–9.

59.	Zähringer J, Schwingshackl L, Movsisyan A, Stratil JM, Capacci S, Steinacker JM, et al. Use of the GRADE approach in health policymaking and evaluation: a scoping review of nutrition and physical activity policies. Implement Sci. 2020;15:1–18.

**NO**

**Reason: Wrong focus**	**EXCLUDE**

**Label:**

- not psychosocial intervention for improving quality of life

**Step 1: Does the study focus on a psychosocial intervention quality of life?**

YES

**YES**

**Step 2: Is intervention targeted to quality of life of elderly people living with HIV?**

**Reason: Wrong population EXCLUDE**

**Label:**

- Not elderly persons living with HIV

**NO**

**YES**

**Reason: Wrong study type**

**EXCLUDE**

**Label:**

- Review
- Commentary
- Opinion
- Letter
- Case series
- Case report/study

**Step 3: Is this a primary study?**

RCTs, Cohort, case-control, cross-sectional studies

**NO**

**SSA countries**

| Angola  Benin  Botswana  Burkina Faso  Burundi  Cameroon  Cape Verde  Central African Republic  Chad  Comoros  Congo  Cote d'Ivoire  Djibouti  Equatorial Guinea  Ethiopia  Gabon  The Gambia  Ghana  Guinea  Guinea-Bissau  Kenya  Lesotho  Liberia | Madagascar  Malawi  Mali  Mauritania  Mauritius  Mozambique  Namibia  Niger  Nigeria  Rwanda  Sao Tome and Principe  Senegal  Seychelles  Sierra Leone  Somalia  South Africa  Sudan  Swaziland  Tanzania  Togo  Uganda  Zaire  Zambia  Zimbabwe |
| --- | --- |

**YES**

**NO**

**Reason: Wrong setting	EXCLUDE**

**Label**: not Sub-Saharan Africa

**Step 4: Was the study conducted in a country in sub-Saharan Africa?**

**(See list of countries in SSA)**

(See list of countries in SSA)

**YES**

**YES**

**TO BE DECIDED**

**Maybe/Unclear**

The protocol was conceived and designed by MA and ADA. MA and LA will conduct the abstract extraction independently and critique the literature. ADA will be third reviewer. NEW and MGS gave insight into the review’s epidemiological aspect and assisted in drafting the manuscript. COY provided the context details and updated the manuscript. MA, LA and ADA critically revised the manuscript for methodological and intellectual content. All authors approve the final manuscript and take responsibility for its content.

**Funding:** None

**Competing interest:** None declared

**Provenance and peer review** Not commissioned; externally peer reviewed.

RE

**INCLUDE**

**NO**

**EXCLUDE**

State reason

**Figure:** Study selection flowchart

**Additional file 1:**	PRISMA-P (Preferred Reporting Items for Systematic Review and Meta-Analysis Protocols) 2015 checklist: recommended items to address in a systematic review protocol*

| **Section and topic** | **Item No** | **Checklist item** |
| --- | --- | --- |
| **ADMINISTRATIVE INFORMATION** | | |
| Title: |  |  |
| Identification | 1a | Identify the report as a protocol of a systematic review |
| Update | 1b | If the protocol is for an update of a previous systematic review, identify as such |
| Registration | 2 | If registered, provide the name of the registry (such as PROSPERO) and registration number |
| Authors: |  |  |
| Contact | 3a | Provide name, institutional affiliation, email address of all protocol authors; provide physical mailing address of corresponding author |
| Contributions | 3b | Describe contributions of protocol authors and identify the guarantor of the review |
| Amendments | 4 | If the protocol represents an amendment of a previously completed or published protocol, identify as such and list changes; otherwise, state plan for documenting important protocol amendments |
| Support: |  |  |
| Sources | 5a | Indicate sources of financial or other support for the review |
| Sponsor | 5b | Provide name for the review funder and/or sponsor |
| Role of sponsor or funder | 5c | Describe roles of funder(s), sponsor(s), and/or institution(s), if any, in developing the protocol |
| **INTRODUCTION** | | |
| Rationale | 6 | Describe the rationale for the review in the context of what is already known |
| Objectives | 7 | Provide an explicit statement of the question(s) the review will address with reference to participants, interventions, comparators, and outcomes (PICO) |
| **METHODS** | | |
| Eligibility criteria | 8 | Specify the study characteristics (such as PICO, study design, setting, time frame) and report characteristics (such as years considered, language, publication status) to be used as criteria for eligibility for the review |
| Information sources | 9 | Describe all intended information sources (such as electronic databases, contact with study authors, trial registers or other grey literature sources) with planned dates of coverage |
| Search strategy | 10 | Present draft of search strategy to be used for at least one electronic database, including planned limits, such that it could be repeated |
| Study records: |  |  |
| Data management | 11a | Describe the mechanism(s) that will be used to manage records and data throughout the review |
| Selection process | 11b | State the process that will be used for selecting studies (such as two independent reviewers) through each phase of the review (that is, screening, eligibility and inclusion in meta-analysis) |
| Data collection process | 11c | Describe planned method of extracting data from reports (such as piloting forms, done independently, in duplicate), any processes for obtaining and confirming data from investigators |
| Data items | 12 | List and define all variables for which data will be sought (such as PICO items, funding sources), any pre-planned data assumptions and simplifications |
| Outcomes and prioritization | 13 | List and define all outcomes for which data will be sought, including prioritization of main and additional outcomes, with rationale |
| Risk of bias in individual studies | 14 | Describe anticipated methods for assessing risk of bias of individual studies, including whether this will be done at the outcome or study level, or both; state how this information will be used in data synthesis |
| Data synthesis | 15a | Describe criteria under which study data will be quantitatively synthesized |
|  | 15b | If data are appropriate for quantitative synthesis, describe planned summary measures, methods of handling data and methods of combining data from studies, including any planned exploration of consistency (such as I^2^, Kendall's τ) |
|  | 15c | Describe any proposed additional analyses (such as sensitivity or subgroup analyses, meta-regression) |
|  | 15d | If quantitative synthesis is not appropriate, describe the type of summary planned |
| Meta-bias(es) | 16 | Specify any planned assessment of meta-bias(es) (such as publication bias across studies, selective reporting within studies) |
| Confidence in cumulative evidence | 17 | Describe how the strength of the body of evidence will be assessed (such as GRADE) |

* It is strongly recommended that this checklist be read in conjunction with the PRISMA-P Explanation and Elaboration (cite when available) for important clarification on the items. Amendments to a review protocol should be tracked and dated. The copyright for PRISMA-P (including checklist) is held by the PRISMA-P Group and is distributed under a Creative Commons Attribution Licence 4.0.

*From: Shamseer L, Moher D, Clarke M, Ghersi D, Liberati A, Petticrew M, Shekelle P, Stewart L, PRISMA-P Group. Preferred reporting items for systematic review and meta-analysis protocols (PRISMA-P) 2015: elaboration and explanation. BMJ. 2015 Jan 2;349(jan02 1):g7647.*

**Additional file 2** PRISMA-P 2020 Flow Diagram*

**Identification of studies via other methods**

**Identification of studies via databases and registers**

Records identified from:

Websites (n = )

Organisations (n = )

Citation searching (n = )

Records removed *before screening*:

Duplicate records removed

(n = )

Records marked as ineligible by automation tools (n = )

Records removed for other reasons (n = )

Records identified from*:

Databases (n = )

Registers (n = )

**Identification**

Records screened

(n = )

Records excluded**

(n = )

**Screening**

Reports not retrieved

(n = )

Reports sought for retrieval

(n = )

Reports sought for retrieval

(n = )

Reports not retrieved

(n = )

Reports excluded:

Reason 1 (n = )

Reason 2 (n = )

Reason 3 (n = )

etc.

Reports assessed for eligibility (n = )

Reports excluded:

Reason 1 (n = )

Reason 2 (n = )

Reason 3 (n = )

etc.

Reports assessed for eligibility

(n = )

Studies included in review

(n = )

Reports of included studies

(n = )

**Included**

* The PRISMA Flow diagram shows the flow of studies from retrieval from electronic databases and other sources to selection of studies for inclusion in the systematic review

**Additional file 3: Hoy Risk of Bias Tool**

| **Risk of bias item** | **Criteria for answers** |
| --- | --- |
| **External validity** | |
| 1. Was the study's target population **a close representation** of the population of interest in relation to relevant variables, e.g. age, sex, occupation, health status or other? | - **Yes (LOW RISK):** The study's target population was a close representation of the national population. - **No (HIGH RISK):** The study's target population was clearly NOT representative of the national population. |
| 2. Was the sampling frame a **true or close representation** of the target population? | - **Yes (LOW RISK):** The sampling frame was a true or close representation of the target population. - **No (HIGH RISK):** The sampling frame was NOT a true or close representation of the target population. |
| 3. Was some form of **random selection** used to select the sample, OR, was a census undertaken? | - **Yes (LOW RISK):** A census was undertaken, OR, some form of random selection was used to select the sample (e.g. simple random sampling, stratified random sampling, cluster sampling, systematic sampling). - **No (HIGH RISK):** A census was NOT undertaken, AND some form of random selection was NOT used to select the sample. |
| 4. Did the study avoid inappropriate exclusions? | - **No (LOW RISK)** - **Yes (HIGH RISK)** |
| **Internal validity** | |
| 5. Was an acceptable case definition used in the study? | - **Yes (LOW RISK):** An acceptable case definition was used. - **No (HIGH RISK):** An acceptable case definition was NOT used. |
| 6. Is the study method for measuring drug prescription shown to have **reliability and validity (if necessary)**? i.e. is there an opportunity for misclassification | - **Yes (LOW RISK):** The method is shown to have minimal misclassification potential - **No (HIGH RISK):** The method is NOT shown to have minimal misclassification potential |
| 7. Was the **same mode of data collection** used for all subjects? | - **Yes (LOW RISK):** The same mode of data collection was used for all subjects. - **No (HIGH RISK):** The same mode of data collection was NOT used for all subjects. |
| 8. Were the **numerator(s) and denominator(s)** for the parameter of interest appropriate? | - **Yes (LOW RISK):** The paper presented appropriate numerator(s) AND denominator(s) for the parameter of interest. - **No (HIGH RISK):** The paper did present numerator(s) AND denominator(s) for the parameter of interest but one or more of these were inappropriate. |
| **Summary item on the overall risk of study bias** | |
| - **LOW RISK OF BIAS:** Further research is very unlikely to change our confidence in the estimate. - **MODERATE RISK OF BIAS:** Further research is likely to have an important impact on our confidence in the estimate and may change the estimate. - **HIGH RISK OF BIAS:** Further research is very likely to have an important impact on our confidence in the estimate and is likely to change the estimate. | |
