# Supplementary material for: Psychosocial interventions and their effectiveness on quality of life among elderly persons living with HIV in Africa South of the Sahara: Systematic review and meta -analysis protocol
Source: PLoS One. 2023 Sep 20;18(9):e0291781. doi: 10.1371/journal.pone.0291781 (PMC10511069; doi:10.1371/journal.pone.0291781)
Supplement: Attachment — Submitted filename: Rebuttal-Psychosocial Interventions QoL_HIV _30 08 2023.docx [file pone.0291781.s005.docx]

PONE-D-23-19414

Effects of Psychosocial Interventions on Quality of Life Among Elderly Persons Living with HIV and AIDS in Sub-Saharan Africa: Systematic Review Protocol

Dear Dr. Felix Bongomin

Thank you for your prompt attention and the opportunity to submit a revised version of our manuscript entitled "Effects of Psychosocial Interventions on Quality of Life Among Elderly Persons Living with HIV and AIDS in Sub-Saharan Africa: Systematic Review Protocol" for consideration for publication in PLOS ONE. We have addressed all comments point-by-point in our response below. The editor’s comments are presented in normal font, and we respond directly to them in bold black font. All changes to the manuscript are highlighted in red font.

Yours sincerely,

Danso-Appiah et al.

**We have addressed all the points raised during the review process in this revised version of the manuscript and used the opportunity to also do minor corrections.**

**Journal requirements:**

1. Please ensure that your manuscript meets PLOS ONE's style requirements, including those for file naming. The PLOS ONE style templates can be found at

<https://journals.plos.org/plosone/s/file?id=wjVg/PLOSOne_formatting_sample_main_body.pdf> and

<https://journals.plos.org/plosone/s/file?id=ba62/PLOSOne_formatting_sample_title_authors_affiliations.pdf>.

**Thank you, we have followed PLOS ONE style requirements, including file naming.**

2. Please amend either the title on the online submission form (via Edit Submission) or the title in the manuscript so that they are identical.

**Thank you, we have made the necessary changes and the title on the submission form and that in the manuscript are the same.**

3. Your ethics statement should only appear in the Methods section of your manuscript. If your ethics statement is written in any section besides the Methods, please move it to the Methods section and delete it from any other section. Please ensure that your ethics statement is included in your manuscript, as the ethics statement entered into the online submission form will not be published alongside your manuscript.

**Thank you, we have moved the ethics statement from after “List of abbreviations” (page 19, last paragraph) to the “Methods” (page 17, first paragraph).**

4. Please review your reference list to ensure that it is complete and correct. If you have cited papers that have been retracted, please include the rationale for doing so in the manuscript text, or remove these references and replace them with relevant current references. Any changes to the reference list should be mentioned in the rebuttal letter that accompanies your revised manuscript. If you need to cite a retracted article, indicate the article’s retracted status in the References list and also include a citation and full reference for the retraction notice.

**We have reviewed the references. Microsoft Excel as suggested by the reviewer has been cited. Also, I replaced the weblink to Rayyan (cited in-text) with the correct bibliographic citation. The total references now stand at 61.**

**Additional Editor comments**

Please find attached manuscript with my comments

[Note: HTML markup is below. Please do not edit.]

[NOTE: If reviewer comments were submitted as an attachment file, they will be attached to this email and accessible via the submission site. Please log into your account, locate the manuscript record, and check for the action link "View Attachments". If this link does not appear, there are no attachment files.]

While revising your submission, please upload your figure files to the Preflight Analysis and Conversion Engine (PACE) digital diagnostic tool, <https://pacev2.apexcovantage.com/>. PACE helps ensure that figures meet PLOS requirements. To use PACE, you must first register as a user. Registration is free. Then, login and navigate to the UPLOAD tab, where you will find detailed instructions on how to use the tool. If you encounter any issues or have any questions when using PACE, please email PLOS at [figures@plos.org](mailto:figures@plos.org). Please note that Supporting Information files do not need this step.

**We have revised Figure 1 accordingly in our manuscript.**

**Reviewer’s comments**

1. Remove AIDS all through the manuscript.

**We have removed AIDS all through the manuscript as suggested.**

2. Use the current recommneded /acceptable terminology to replace SSA.

**We have used the current recommended teminology as suggested - Africa South of the Sahara.**

3. *a systematic review and meta-analysis protocol.*

**We have revised the title of the protocol accordingly.**

4. Why this start date?

**Searches will be conducted to retrieve relevant studies without time (date of publication) limits. This has now been stated in the manuscript.**

5. Consider up to 31st December 2023.

**Thank you, our final search date has now been changed from “30^th^ September 2023” to “31^st^ December 2023”.**

6. Human Immune deficiency virus (HIV) and Acquired Immune Deficiency Syndrome (AIDS) Check how HIV is written in full.

**Thank you, the authors have acknowledged this typographical error and have corrected the full meaning of HIV as Human Immunodeficiency Virus.**

7. Delete “AIDS” all through the text.

**Thank you for this suggestion, the term AIDS has been deleted from the whole manuscript except for the name of one of the affiliations which cannot be changed.**

8. The rationale for this systematic review

**Thank you, we have deleted the heading “Rationale for this systematic review”.**

9. Microsoft Excel

**We have provided citation to “Microsoft Excel.**

10. SUGGEST POSSIBLE LIMITATIOSN AND HOW YOU WILL OVERCOME THEM

**Thank you. We have included in the manuscript the anticipated study limitations and the strategies we will adopt in addressing them.**

11. SUGGEST IMPLICATIONS OF THE ANTICIPATED STUDY FINDINGS

**Thank you. We have included a section on the implication of the review findings in the manuscript.**
